# Enhanced Robot Motion Block of A-Star Algorithm for Robotic Path Planning

**DOI:** 10.3390/s24051422

**Published:** 2024-02-22

**Authors:** Raihan Kabir, Yutaka Watanobe, Md Rashedul Islam, Keitaro Naruse

**Affiliations:** 1 Department of Computer Science and Engineering, University of Aizu, Aizu-Wakamatsu 965-8580, Japan; raihan.kabir.cse@gmail.com (R.K.); naruse@u-aizu.ac.jp (K.N.); 2 Division of Computer Vision and AI, Department of R&D, Chowagiken Corp., Sapporo 001-0021, Japan; rashed.cse@gmail.com

**Keywords:** A* algorithm, adaptive cost function, BFS, Dijkstra, DFS, TWA*, path planning, robot motion block (RMB), ROS, SLAM, Gazebo simulator

## Abstract

An optimized robot path-planning algorithm is required for various aspects of robot movements in applications. The efficacy of the robot path-planning model is vulnerable to the number of search nodes, path cost, and time complexity. The conventional A-star (A*) algorithm outperforms other grid-based algorithms because of its heuristic approach. However, the performance of the conventional A* algorithm is suboptimal for the time, space, and number of search nodes, depending on the robot motion block (RMB). To address these challenges, this paper proposes an optimal RMB with an adaptive cost function to improve performance. The proposed adaptive cost function keeps track of the goal node and adaptively calculates the movement costs for quickly arriving at the goal node. Incorporating the adaptive cost function with a selected optimal RMB significantly reduces the searches of less impactful and redundant nodes, which improves the performance of the A* algorithm in terms of the number of search nodes and time complexity. To validate the performance and robustness of the proposed model, an extensive experiment was conducted. In the experiment, an open-source dataset featuring various types of grid maps was customized to incorporate the multiple map sizes and sets of source-to-destination nodes. According to the experiments, the proposed method demonstrated a remarkable improvement of 93.98% in the number of search nodes and 98.94% in time complexity compared to the conventional A* algorithm. The proposed model outperforms other state-of-the-art algorithms by keeping the path cost largely comparable. Additionally, an ROS experiment using a robot and lidar sensor data shows the improvement of the proposed method in a simulated laboratory environment.

## 1. Introduction

In the field of robotics, there are several challenging tasks; among these, one of the most important tasks is path planning, which determines how to reach a desired goal position. In this research area, the main objective is to develop an efficient path-planning algorithm by enhancing its performance in terms of searching time complexity, path cost, and search area [1]. As the application area of robot usage is increasing rapidly, the number of ongoing studies is also increasing in this area [2]. Generally, path-planning algorithms always search for the target position from the current position of the robot by keeping the overall path as short as possible. There are many path-planning algorithms for minimizing the path cost and finding the shortest path; however, for a path-planning algorithm, the importance of the three main factors, time complexity, path cost, and search area, mainly depends on the robot application [1]. Nevertheless, considering these factors, a general and efficient path-planning algorithm fits most robot applications.

Generally, path-planning algorithms are categorized into two groups based on the surroundings of the robot and the amount of known environmental information: global path planning and local path planning [3,4]. A robot with global path planning needs to partially know the environmental information, such as the map of the environment required to calculate the optimal route from the start position to the goal position. How well the algorithm partially knows the obstacle map and the effectiveness of the algorithm are evaluated by its ability to find a valid path if one exists [5,6]. In contrast, local path planning requires the robot to search its surroundings using sensors and find a path to reach the goal in an environment with unknown information [5]. As a result, local path-planning algorithms are typically more time-consuming and complex and may require the use of techniques such as segmentation and object detection to avoid obstacles [7]. Some commonly used local path-planning algorithms include the artificial potential field method, fuzzy logic method, and dynamic window method. In some cases, an ArUco marker-based searching system can make the process easier and more efficient [8]. In the global path-planning field, various algorithms are available, such as rapidly exploring random trees (RRTs), graph searching, tangent graph-based planning, and predefined path optimization. To test performance, simulators such as Gazebo or the ROS (Robot Operating System) platform provide the required features [9]. Our research focuses on the area of graph-based searching algorithms and performance testing in a simulator.

Graph-based searching algorithms are extensively used and considered highly effective in the field of global path planning. Some popular examples of graph-based search algorithms include A*, Dijkstra, depth-first search (DFS), and breadth-first search (BFS). Among these algorithms, A* is particularly notable for its completeness, efficient search area, and ability to use heuristics [10]. A* is a path-finding algorithm developed on the basis of the Dijkstra algorithm and is designed for use on weighted graphs, such as grid maps with obstacles. The algorithm works by constructing a tree of paths from the starting node and adding nodes as it searches the graph until it reaches the goal node or exhausts all options [10]. The final result is the shortest path found in the tree of searched paths. To search the graph or grid map, the algorithm requires an RMB to navigate the surrounding environment. The research goal is to develop an optimal RMB to improve the overall performance of the A* algorithm.

The robot motion block is a crucial element in path-planning algorithms such as A*, as it determines how the robot will search the cells surrounding its current position as it moves toward its destination [11]. The RMB is a matrix that specifies the cells that the robot should search in each iteration to find the goal node, starting from the start node. The performance and efficiency of the algorithm are significantly affected by the size of the motion block matrix, as it determines the search area of the robot in each iteration [12]. An inefficient motion block results in the robot searching for a larger number of cells to find the goal, increasing the time and space complexity. In the conventional approach, the motion block matrix comprises eight surrounding cell coordinates for the robot to search [11]. In each iteration, the A* algorithm adds the information about these eight cells to its search queue and continues to add cells proportionally until it reaches the goal or exhausts all options. This approach necessitates a significant amount of searching time complexity and many search nodes. Thus, keeping these limitations in mind, this study proposes an optimal RMB with an adaptive cost function for robot movement to improve performance. The proposed method considers eight neighboring coordinates with different searching distances and puts only the end node in the search queue, significantly reducing the number of search nodes and time complexity with a small path cost increase. As the path cost increases and the number of search nodes and time complexity decrease with different sizes of motion block, a formula is also proposed to decide the optimal path among different distances. Using the proposed method, the A* algorithm can find the goal position by avoiding obstacles with significantly improved overall performance. A comparison study with the conventional A* and similar state-of-the-art path-planning algorithms and experiments on an ROS platform validates the significant improvement of the proposed model. In addition, this study discusses the effectiveness, drawbacks, and applicability of the proposed approach in different application areas.

In summary, the key contributions of this research include:

- This paper proposes an adaptive cost function for robot movement, which keeps track of the goal node, adaptively calculates the movement costs for quickly getting to the goal node, and reduces the number of search nodes.
- An optimal RMB with an adaptive cost function is proposed to improve the performance of robot navigation by significantly reducing the searches of less impactful and redundant nodes. The reduced number of search nodes significantly improves the time complexity and the overall performance of the A* algorithm.
- In this paper, a comprehensive experiment is conducted by customizing a large open-source dataset, including various types of grid maps, various map sizes, and several sets of source-to-destination nodes. This experiment validates the proposed model in terms of efficiency, robustness, and scalability.
- Also, conducting experiments using the ROS platform and Gazebo simulator, this paper adds empirical evidence, strengthening the contributions and demonstrating the generalizability of the proposed model in real-world scenarios.
- The quantifiable results, revealing substantial improvements in the number of search nodes and time complexity, distinctly position the proposed model as an advancement over conventional A* and other state-of-the-art algorithms.

The remainder of this paper is structured as follows: Section 2 provides an overview of related works, Section 3 presents the details of the proposed model, Section 4 offers the discussion and results of the experiments conducted, and Section 5 concludes the study.

## 2. Related Work

In the field of robotics, path-planning algorithms have been extensively investigated by researchers to improve their efficiency and reduce search costs. Despite considerable progress in this area, the performance of these algorithms still has room for improvement, and researchers are still exploring new ways to enhance their reliability and efficiency. This is particularly important in robotics applications, where fast and accurate path planning is essential for the safe and effective operation of robots that can handle increasingly complex environments and tasks while maintaining a high performance level.

In one study, Li Changgeng et al. proposed a new global path-planning algorithm called bidirectional alternating search A* (BAS-A*) for mobile robots [13]. Their algorithm combines the best features of traditional A* and bidirectional search algorithms to generate efficient and optimal paths. By utilizing a bidirectional alternating search strategy, the algorithm improves search efficiency by searching forward and backward path lists until they intersect. The algorithm addresses issues such as long calculation times, large turning angles, and unsmoothed paths in large task spaces. They introduced weighted heuristic functions, a filtering function for path nodes, and Bézier curves to ensure smooth and optimized path planning [14]. Simulation results demonstrated the algorithm’s efficiency and smoothness compared to other algorithms. Practical validation was also attempted on the TurtleBot3 Waffle Pi mobile robot [15].

Meanwhile, Szczepanski et al. presented a hybrid approach for the global path planning of mobile robots in variable workspaces [16]. Their approach comprised an offline global path optimization algorithm using the artificial bee colony algorithm and an online path-planning scheme that uses a graph created from the control path points generated in the offline part [17]. The online part uses the Dijkstra algorithm to find the shortest path [18]. Their proposed approach aimed to generate optimal paths for mobile robots in variable workspaces which are feasible even with new obstacles. Their approach was tested in the Matlab/Simulink environment and achieved a good result.

In another study, Sánchez–Ibáñez et al. conducted a comprehensive review that provided an overview of path-planning algorithms for autonomous mobile robots [19]. They highlighted the importance of autonomous capabilities in enhancing economic and safety aspects. With numerous path-planning algorithms available, the authors aimed to classify and analyze these algorithms, particularly for autonomous ground vehicles [20]. Their review covered environmental representation, robot mobility, dynamics, and various path-planning categories. The study served as a valuable resource for understanding the research conducted on path-planning algorithms, aiding in the selection of appropriate algorithms for specific requirements.

Tripathy et al. proposed a collision-free navigation scheme for mobile robots in a grid environment [21]. This research scheme combines a radio frequency identification method for robot localization, a hybrid approach for path planning, and a predefined decision table for navigation [22,23]. The algorithm has two stages: construction of the virtual world and generation of the optimal shortest path. The performance of the algorithm was evaluated in different grid-based environments with and without obstacles [24]. The results showed that the robot explored fewer cells to find the shortest path when there were no obstacles. However, in environments with obstacles, the number of turns in the shortest path was always less than that when exploring the entire virtual world.

In another investigation, Ou et al. address challenges inherent in traditional A* algorithms in terms of excessive turning points and sluggish search speed. They present an enhanced approach, integrating lidar and an IMU on the mobile robot platform [25]. The method utilizes Hdl_graph_slam mapping to construct a two-dimensional grid map and ROS platform. Path planning is executed through an improved A* algorithm, incorporating strategies for path smoothing and safety. The algorithm achieves a 13% reduction in average path search time and an 11% reduction in average search extension nodes, effectively mitigating the issues of excessive turning points and slow search speed associated with traditional A* algorithms. In a related study, Abbyasov et al. outline a methodology for prototyping an authentic office environment within the Gazebo simulator, utilizing the Blender modelling suite for high-quality 3D model generation [26]. The validation of their developed virtual environment involves conducting lidar-based SLAM (simultaneous localization and mapping) tasks with a mobile robot and testing them using advanced Gazebo actors.

Separately, Chen et al. introduced a three-neighbor search A* algorithm combined with the artificial potential field method to overcome irregular forward direction obstacles and guide robot movement [27,28]. Their method demonstrated significant improvements by reducing the number of search nodes, search time, and path length by 88.85%, 77.05%, and 5.58%, respectively. Saeed et al. presented the boundary node method, an offline path-planning approach that generated collision-free paths for mobile robots [29]. Their method utilized a nine-node quadrilateral element and a potential function to guide robot movement, generating initial collision-free paths quickly and safely. They also employed a path enhancement method to further optimize the path length. The simulation results validated the effectiveness of their proposed methods. In a different approach, Ichter et al. introduced latent sampling-based motion planning, combining recent control advances with techniques from sampling-based motion planning (SBMP) [30,31]. Their methodology involved learning a plannable latent representation using autoencoding, dynamics, and collision-checking networks. The learned latent RRT algorithm demonstrated global exploration capabilities and generalization to new environments, as showcased in visual planning and humanoid robot path-planning problems [32]. These studies provide valuable insights into different path-planning approaches that helped us to address the limitations of traditional A* algorithms and offer improved performance and adaptability for various robotic systems. Table 1 presents a summary of the related works in the area of path-planning methods and the method of validating the performance improvement, where “√“ indicates the consideration of that criteria in the related work and “-“ indicates criteria that are not considered.

## 3. Proposed Model

**Figure 1:**
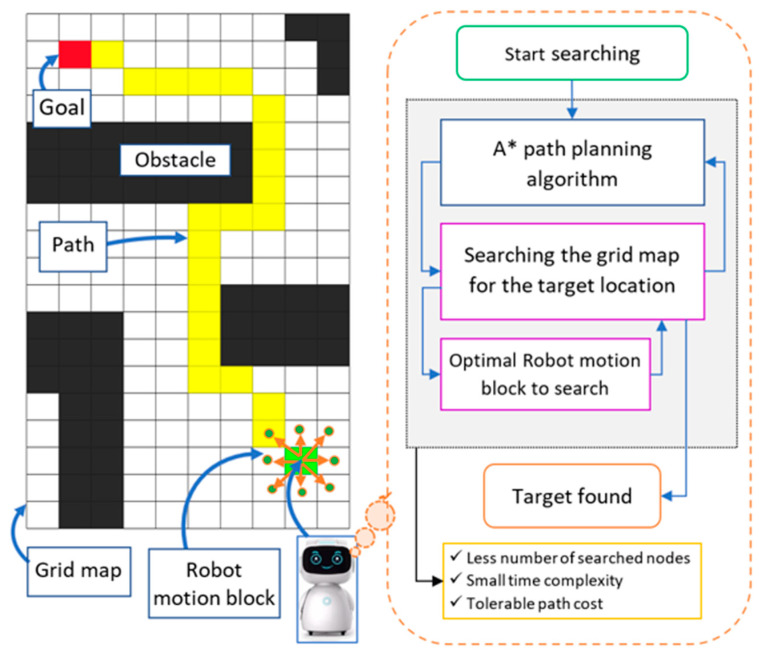
Block diagram of our proposed model.

The A* algorithm is most effective in finding the shortest path due to its heuristic search technique. The conventional A* algorithm takes a long time to search and checks a large number of cells when searching for a goal because of an inefficient RMB. For a robot to reach its destination, it needs to find the shortest path from the starting position to the goal by checking a minimum number of search nodes with low time complexity. The proposed RMB helps to significantly reduce those two criteria and improves the overall performance of the A* path-planning algorithm while avoiding obstacles. The block diagram in Figure 1 illustrates the proposed model, which includes a robot path-planning algorithm with an efficient RMB to search for the optimal path. In this model, the robot examines the neighboring grid cells using the proposed optimal RMB, which significantly improves the performance of the path-planning algorithm and increases its usability in real-time. The proposed model was experimented on using various grid maps that contain different obstacle environments and starting and goal positions.

### 3.1. A* Path-Planning Algorithm with Proposed RMB

**Figure 2:**
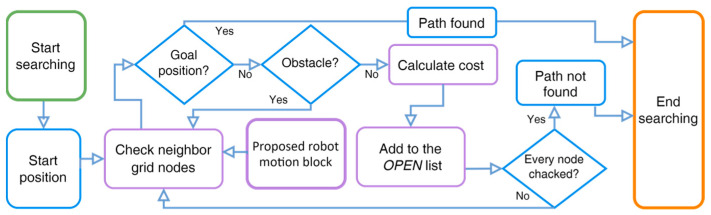
Block diagram of the A* path-planning algorithm with the proposed optimal RMB.

Currently, there exist numerous algorithms for searching paths and traversing graphs, one of which is A*. It is extensively recognized for its efficiency, being both optimal and complete. A* is classified as a best-first search or informed search algorithm that operates on a weighted graph. Its objective is to find a goal node, beginning from a specified starting node, with the smallest amount of travel cost and time. The algorithm creates a tree of search nodes, beginning from the starting node, and continues until the search terminates, which can either be the discovery of the goal node or no discovery at all. The A* algorithm uses an heuristic function, $h\left(x\right),$ to estimate the cost, $c\left(s,s^{\prime }\right),$ to reach the goal node from the current node [33], where $h\left(x\right)\leq c\left(x,x^{\prime }\right)+h\left(x^{\prime }\right)$ and $c\left(x,x^{\prime }\right)$ is the cost of an individual node $x\neq x_{goal}$ and any successor $x^{\prime }$. This heuristic should be both admissible (it should never overestimate the actual cost to reach the goal) and consistent (the cost from one node to another should not be more than the cost to move directly from the first node to the goal node). The heuristic function for the grid map is the Euclidean distance between the current node and the goal node. The algorithmic workflow of the A* algorithm, using the proposed model, shown in Figure 2, is as follows:

- To begin the A* algorithm with the proposed robot motion block, initialize the start node $\left(S_{start}\right)$ with a cost of 0 and add it to an OPEN list of nodes to be considered for expansion.
- Check the neighboring grid nodes for the goal node ${(S}_{goal})$ with the proposed robot motion block explained in Section 3.2. If the current node is an obstacle node ${(S}_{obstacle})$, ignore it, and add only the end nodes in the OPEN list. While the OPEN list is not empty, select the node with the lowest total cost, $Cost\left(C\right),$ and remove it from the OPEN list.
- $Cost(C)=min\left\{OPEN,\;h\left(S_{goal},\;OPEN\right)\;\right\}$
- Here, $h\left(S_{goal},OPEN\right)$ denotes an heuristic function that uses Euclidean distance, $d\left(p,q\right)=\sqrt{{\left(q_{1}-p_{1}\right)}^{2}+{\left(q_{2}-p_{2}\right)}^{2}}$
- If the selected node is the goal node, stop the search and return the optimal path. Generate all possible successors of the selected node and calculate their costs and heuristic values.
- For each successor node, update its cost and heuristic value if it has not already been visited or has a higher cost. Add each updated successor node to the OPEN list if it is not already in the list.
- If a successor node is already in the OPEN list, update its cost and heuristic value if the new values are lower than the previous values. If a successor node is already in the visited list (i.e., has already been expanded), ignore it.
- If all nodes in the motion block cells have been checked, return to step II and repeat the steps until every node on the grid map has been checked.
- Terminate the algorithm when the goal node is found or the OPEN list is empty.

### 3.2. Proposed Robot Motion Block with an Adaptive Cost-Function

In a grid-based search, robots must search the cells around them to find the goal node in the smallest number of cells possible. The robot uses a block of cells, called the robot motion block or motion kernel, to search for its neighboring nodes. However, the conventional RMB used by A* leads to a higher number of search cells and a longer search time. The proposed motion block, which surrounds the robot with eight neighboring cells, aims to minimize the number of search cells and the time taken to find the goal position while maintaining a comparable path cost. The

(1) $$Motion\;block=\left[\begin{array}{ccc} n & 0 & CC\left(d,C\right)\\ 0 & n & CC\left(d,C\right)\\ -n & 0 & CC\left(d,C\right)\\ 0 & -n & CC\left(d,C\right)\\ -n & -n & DC\left(d^{\prime },C\right)\\ -n & n & DC\left(d^{\prime },C\right)\\ n & -n & DC\left(d^{\prime },C\right)\\ n & n & DC\left(d^{\prime },C\right) \end{array}\right]$$

Here,

n=1,2,3,…,N
CC = Cardinal movement cost.
DC = Diagonal movement cost.
d = The cost associated with cardinal movements.
$d^{\prime }$ = The cost associated with diagonal movements.
C = Adaptive cost.

**Figure 3:**
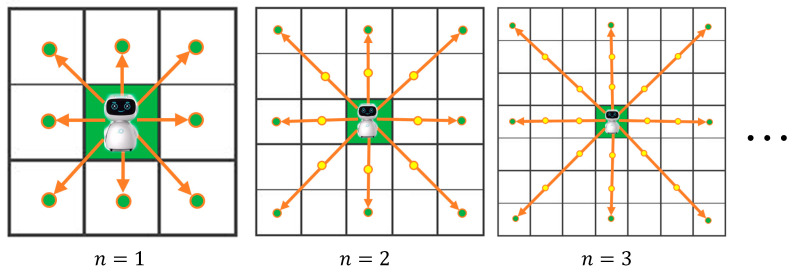
Block diagram of the A* path-planning algorithm with the proposed optimal RMB. Orange arrows = search directions, green and yellow = search nodes.

The proposed RMB is represented by a matrix as Equation (1), where *n* is the size of the RMBs. Figure 3 provides a visual representation of the proposed RMBs.

The RMB has eight neighboring cell directions, including up, down, left, right, up-right, up-left, down-right, and down-left. The costs of the four cardinal and diagonal directed points are represented by $CC\left(d,C\right)$ and $DC\left(d^{\prime },C\right)$, respectively. These costs are calculated using Equations (2)–(4):

(2) $$\begin{array}{c} d_{i}=1,\;\;\;\;1\leq i\leq 4\;\;\mathrm{and}\;{d^{\prime }}_{i}=\sqrt[{2}]{2},\;\;\;5\leq i\leq 8\\ C^{\prime }\left({Ccn}_{j},{cn}_{j},\;q_{i}\right)={Ccn}_{j}+\sqrt[{2}]{{\left({qx}_{i}-{cnx}_{j}\right)}^{2}+{\left({qy}_{i}-{cny}_{j}\right)}^{2}}\\ Subject\;to,1\leq j\leq N;1\leq i\leq 8;cn=Current\;node,\\ \;\;Ccn=Cost\;of\;cn,\;\;q=\mathrm{Nodes}\;\mathrm{of}\;\mathrm{motion}\;\mathrm{matrix}. \end{array}$$

(3) $$\begin{array}{c} C\left(C^{\prime },{gn}_{i},\;q_{i}\right)=C^{\prime }+\left(\sqrt[{2}]{{\left(gnx_{i}-{qx}_{i}\right)}^{2}+{\left({gny}_{i}-{qy}_{i}\right)}^{2}}\right)*\alpha \\ \;\;Subject\;to,gn=Goal\;node,and\;0.001\leq \alpha \leq 0.009 \end{array}$$

(4) $$\begin{array}{c} CC\left(d,\;C\right)=d*C,\\ DC\left(d^{\prime },\;C\right)=d^{\prime }*\;C, \end{array}$$

To determine the CC and DC costs of the nodes of the motion matrix, an adaptive cost function, C, is formulated, which is multiplied by the costs associated with cardinal d and diagonal $d^{\prime }$ movements, as shown in Equation (4). In this adaptive cost function, first, the current node’s cost, Ccn, is summed with the Euclidian distance between the current node, cn, and the node of motion matrix, q, to obtain the absolute differences in the x and y coordinates. The cost, $C^{\prime }$, is determined as shown in Equation (2). Next, $C^{\prime }$ is summed with the Euclidean distance between the node of motion matrix q and goal node gn by multiplying with a constant of α in Equation (3). Here, α is used to increase the importance of the distance value between each of the motion matrix nodes and the goal node. This helps the adaptive cost function to keep track of the goal node with each node of the motion matrix and reduce the number of searches to find the goal node. The optimal range of α is between 0.001 and 0.009 after testing on a large number of maps. However, α=0.007 was used in this paper, which provided the best results.

### 3.3. Optimal Robot Motion Block Selection

The proposed robot motion block has different sizes (n). The output results for each motion block differ. However, a general optimal RMB is essential for any robotic application. To obtain the optimal one from different RMB sizes, a selection formula is proposed as Equations (5)–(7).

(5) $$Ra{\left(A_{i}\right)}_{j}=R\times \frac{{A(x}_{j})-{\left(A\right)}_{min}}{{\left(A\right)}_{max}{-\left(A\right)}_{min}}$$

(6) $${{Ra}^{\prime }\left(Ra\right)}_{j}=\frac{\sum_{i=1}^{p}{Ra(x}_{j})}{p}$$

Subject to,1≤i≤p,1≤j≤n,R=1000,p=3

(7) $$\begin{array}{c} ORMB\left(Ra^{\prime }\right)=argmin\left(\frac{\sum_{k=1}^{q}Ra\prime (x_{j})}{q}\right)\\ Subject\;to,1\leq k\leq q,\;\;q=5\;(\mathrm{map}\;\mathrm{types}) \end{array}$$

Here, A denotes the array of n elements, and each element contains the mean of the output data, which is experimented on in the dataset containing $\left\{1,\ldots ,N\right\}$ data points based on the RMB size. i denotes parameters and $A\left(x_{j}\right)$ denotes individual output array elements for each RMB size, where $\left\{x_{j},j=1,\ldots ,n\right\}$. To optimize the efficiency of the path-planning algorithm, this study considered three p parameters, namely, path costs, the number of search nodes in the grid map, and time complexity. Next, to measure the importance of the p parameters, each data array is normalized into a range of R=1000 by weighting equally using Equation (5) and achieves a ranged array, Ra. After that, each indexed data point from all resultant data arrays is summed and subscribed by the total number of arrays, which in this study is three, to obtain $Ra^{\prime }.$ Equation (6) was used to calculate $Ra^{\prime }$. Finally, the optimal RMB is determined by selecting the minimum value of the final resultant data array for each RMB. Equation (7) was used to select the optimal RMB, ORMB.

## 4. Experimental Results and Discussion

The experimental outcomes justify the validity and demonstrate the effectiveness of the proposed model. The following subsections describe the experimental results of the proposed optimal RMB for the A* algorithm. First, the preprocessing steps of the dataset with different types of grid maps are described. After that, the experimental results using the prepared dataset show the effectiveness of the proposed model.

### 4.1. Grid Map Preparations Using the Dataset

#### 4.1.1. Dataset

**Figure 4:**
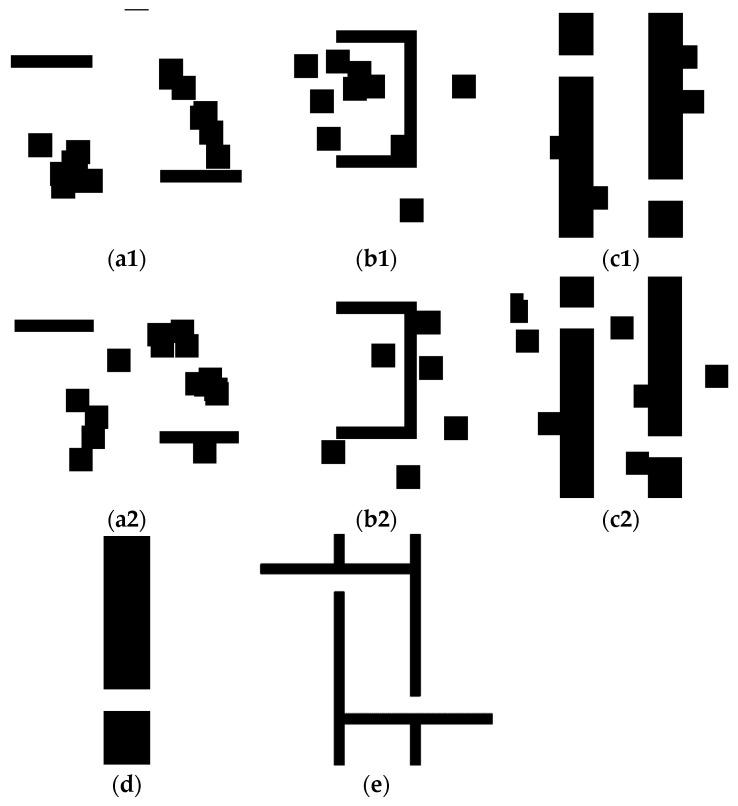
Sample images of grid maps from the dataset (**a1**,**a2**) forest, (**b1**,**b2**) bugtrap_forest, (**c1**,**c2**) gaps_and_forest, (**d**) alternating_gaps, and (**e**) mazes.

To validate the proposed approach, a collection of grid maps is essential for the experiment on the proposed RMB of the A* path-planning algorithm. This experiment uses a benchmarked online public repository dataset named “motion_planning_datasets” [34,35]. There are eight types of grid maps in this dataset. Each grid map type contains thousands of PNG format images with a resolution of 201 × 201. Five types of grid maps of planning environments are used among these eight types: alternating_gaps, forest, bugtraq_forest, gaps_and_forest, and mazes. For validation purposes, 4000 grid maps’ data are used in this research, with 800 grid maps’ data for each type. Figure 4 shows some sample images of these grid maps from the dataset.

#### 4.1.2. Grid-Map Preparation

**Figure 5:**
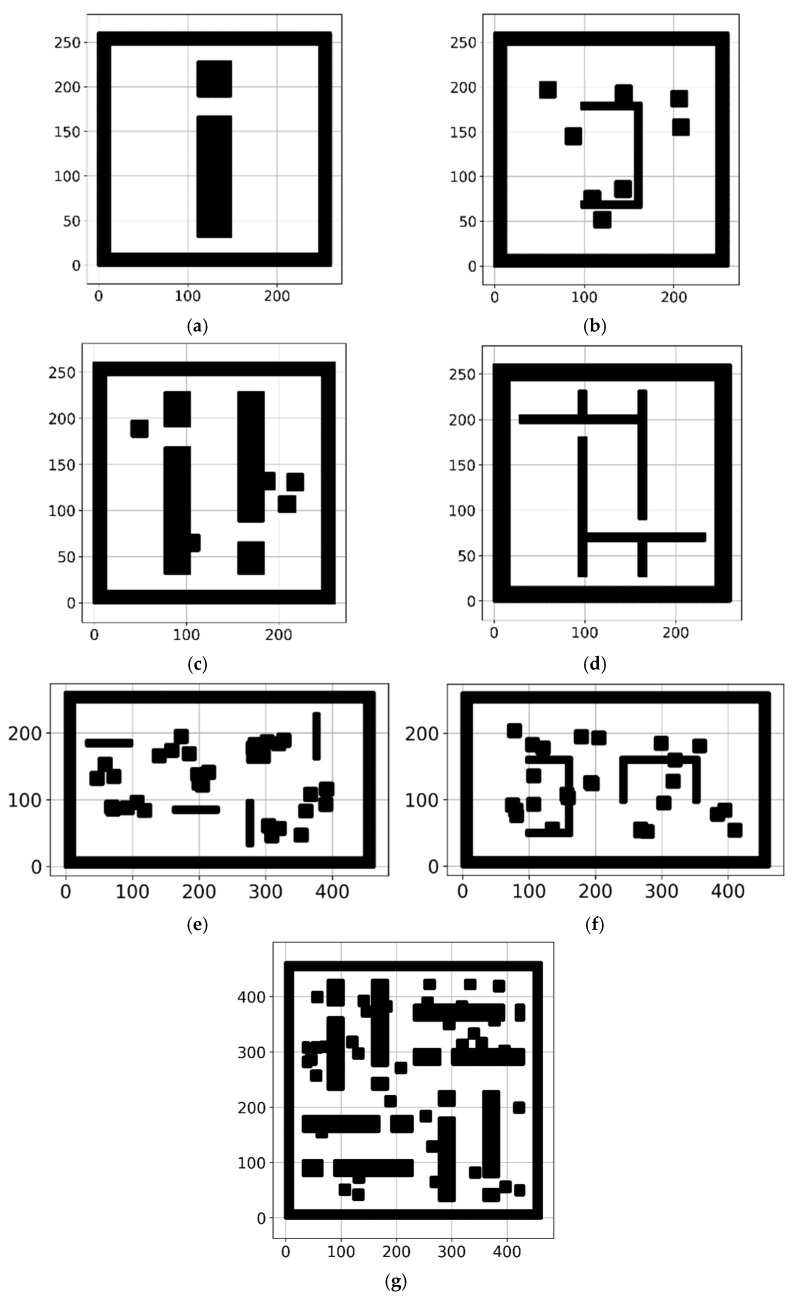
Prepared grid maps from the dataset (261 × 261): (**a**) alternating_gaps, (**b**) forest, (**c**) gaps_and_forest, and (**d**) mazes; (261 × 462): (**e**) forest, (**f**) bugtrap_forest; (462 × 462): (**g**) gaps_and_forest. White = free cells and black = obstacle cells.

The dataset described in the previous subsection has some limitations, such as all grid map data points being images in PNG format rather than the matrix format required for the experiment. For the robot not to leave the grid map, a border surrounding the grid map is essential, but there is no border in the dataset images, and they also have no start or endpoint information. To validate the efficiency, robustness, scalability, and generalization of the proposed method, it needs to be tested on different dimensional grid maps, but the dataset images have a diminution of 201 × 201. To overcome these limitations and fit the dataset with the experiment, the dataset images are preprocessed and the grid maps are prepared for the experimental format, as depicted in Figure 5.

Firstly, three different dimensional types of grid map images are prepared: 261 × 261, 462 × 261, and 462 × 462. To prepare a 462 × 261 dimensional map, two same types of dataset map images are added side by side, and for a 462 × 462 dimensional map, four of the same types of dataset map images are added together. In this way, 200 maps are obtained for 402 × 402, 400 for 402 × 201, and 800 for 201 × 201, as 800 map images are considered from the dataset.

Next, two borders surrounding the dataset grid map images with 15 pixels each are added, making the grid map image dimensions 261 × 261, 462 × 261, and 462 × 462. Among these two borders, the outer border is black obstacle pixels, and the inner border is white obstacle-free pixels. Next, these modified map images are split into grid nodes containing information about each node with and without obstacles to change them to a matrix format. Finally, the grid maps with the required format are obtained to validate the proposed model.

#### 4.1.3. Source and Destination Selection

**Figure 6:**
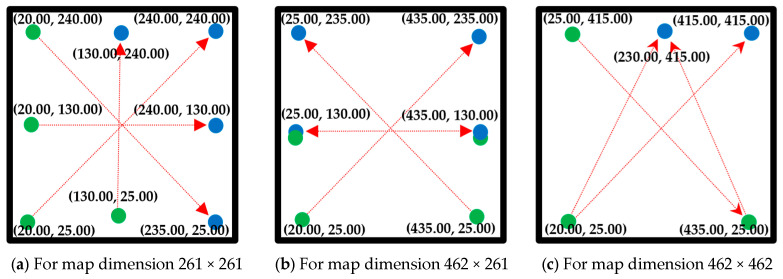
(**a**–**c**) show the source-to-destination direction for three dimensions’ maps. Blue circle = destination, green circle = source, and red arrow = path direction.

For the experiment, four sets of different sources and destinations are selected for each of the three-dimensional maps, as shown in Figure 6. The red arrow represents the direction from the source (depicted as the green circle) to the destination (represented by the blue circle). In addition, besides the blue and green circles, source and destination coordinates are provided. For the map with dimensions 261 × 261, 200 maps are experimented with for each direction, as 800 maps are prepared for this dimensional map. Similarly, 100 maps are used for each direction in the case of the 462 × 261 dimensional map, and 50 maps are employed for the 462 × 462 dimensional maps.

### 4.2. Experimental Results of the Proposed Model

This paper shows the experimental results using the preprocessed 4000 grid maps of five different types for the proposed optimal RMB of the A* algorithm. First, the experiment for the proposed model demonstrates the effectiveness of the proposed RMB by minimizing the number of search nodes and time complexity while maintaining the minimum path cost from the start position to the target position. Then, the selection of the optimal RMB ensures algorithmic optimality by considering the three major criteria: the number of search nodes, time complexity, and path cost. In this paper, two decimal places are presented for all floating values except for the parameter of required time in seconds to show the differences, as in some cases, those values are very small. Also, the best results are presented with bold font in tables. Finally, a comparison between the proposed model and other similar algorithms shows the effective performance of the proposed optimal RMB.

#### 4.2.1. Comparison between Conventional A* and the Proposed Method

**Figure 7:**
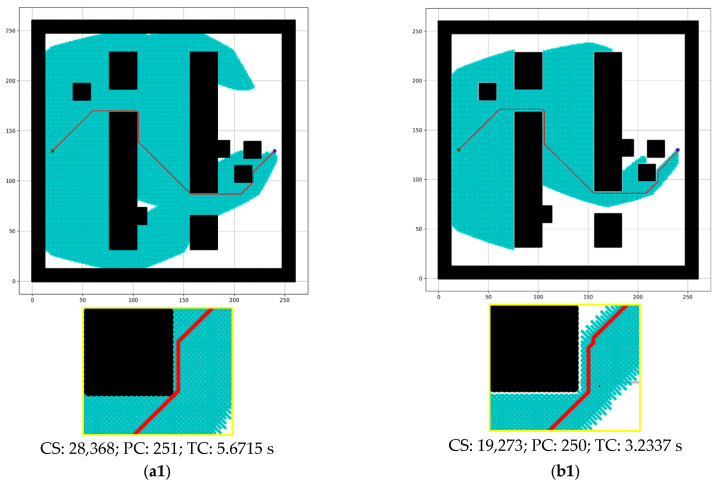

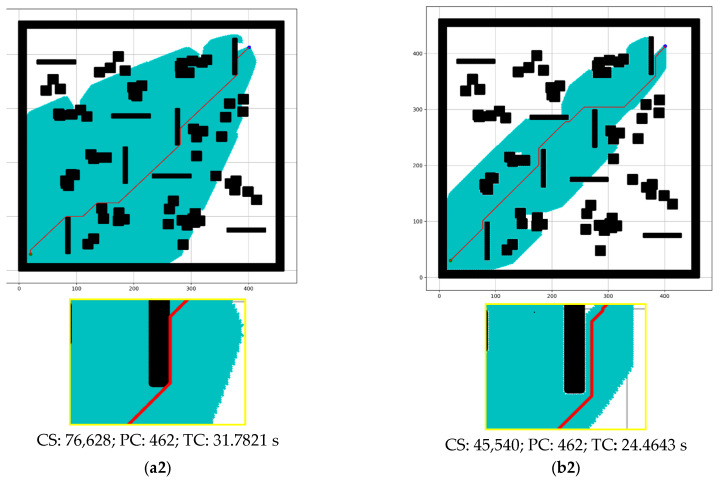
Simulated results for the comparison between (**a1**,**a2**) conventional A* and (**b1**,**b2**) the proposed method for RMB n = 1. Here, CS = number of nodes searched, PC = path cost, TC = time complexity. White = free cell, black = obstacle cell, green and blue circle = start and goal point, red line = found path, and cyan cross = cell searched.

The proposed method performs better than the conventional A* algorithm by introducing the adaptive cost function and RMBs. In Table 2, the comparison shows the improvement for RMB *n* = 1. Figure 7 shows a sample simulated result of this comparison. of this com However, this paper shows further experiments for other values of RMB.

Table 2 shows the cumulative experimental results conducted using the dataset containing five types of grid maps. The proposed method reduces the time complexity by 29.05%, the number of search nodes by 32.58%, and the path cost by 0.04% compared to the conventional A* algorithm, calculated using Equation (8). This is because of the effectiveness of the adaptive cost function described in Section 3.2, which keeps track of the goal node and calculates the robot movement cost adaptively, reducing the number of search nodes and time complexity. However, when n increases, only the end node is placed in the next search list of the search distance, which reduces the number of search nodes and time complexity even more.

(8) $$\begin{array}{c} \mathrm{IE}=\frac{\frac{\sum_{j=1}^{m}x_{j}}{m}-\frac{\sum_{j=1}^{m}y_{j}}{m}}{\frac{\sum_{j=1}^{m}x_{j}}{m}}\times 100,\\ Subject\;to,\;\;\;\;1\leq j\leq m,m=3\;(as\;three\;dimentional\;maps\;are\;considered) \end{array}$$

Here,

IE= Impact Evaluation
x = Value of conventional A*
y = Value of proposed model

#### 4.2.2. Experimental Results Using Different Sizes (n) of the Robot Motion Block

**Figure 8:**
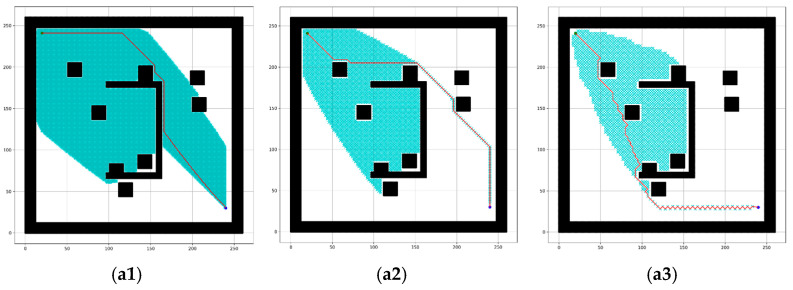

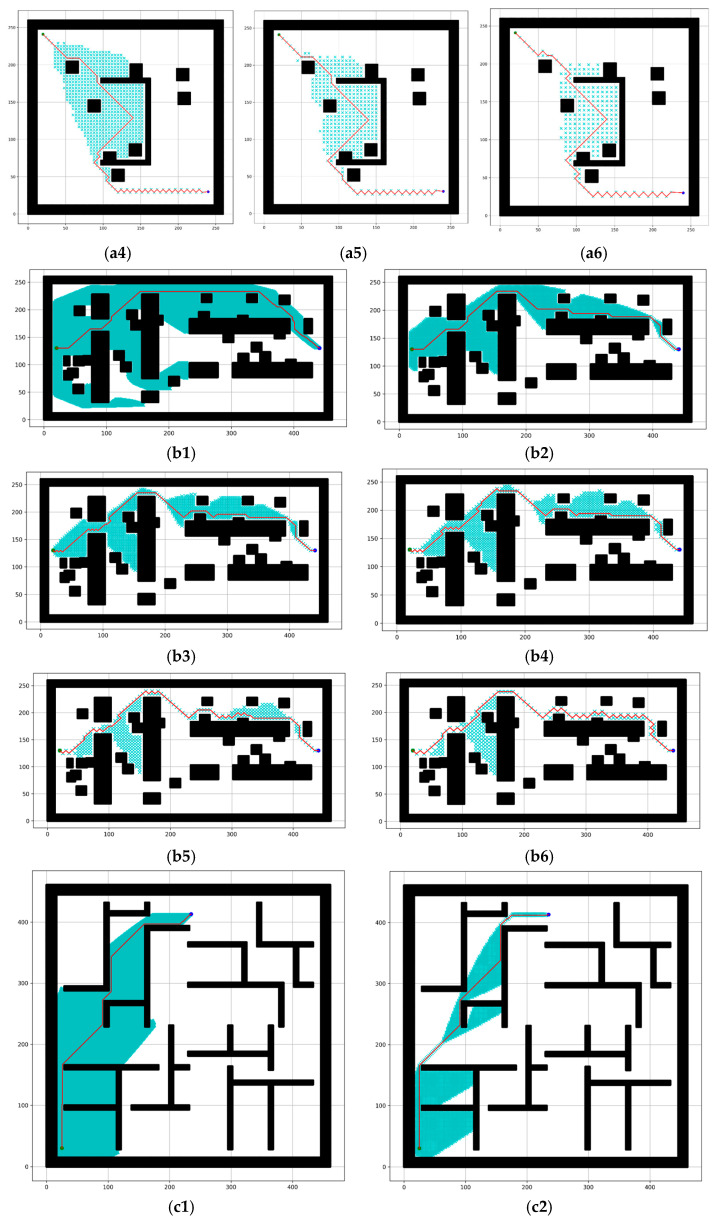

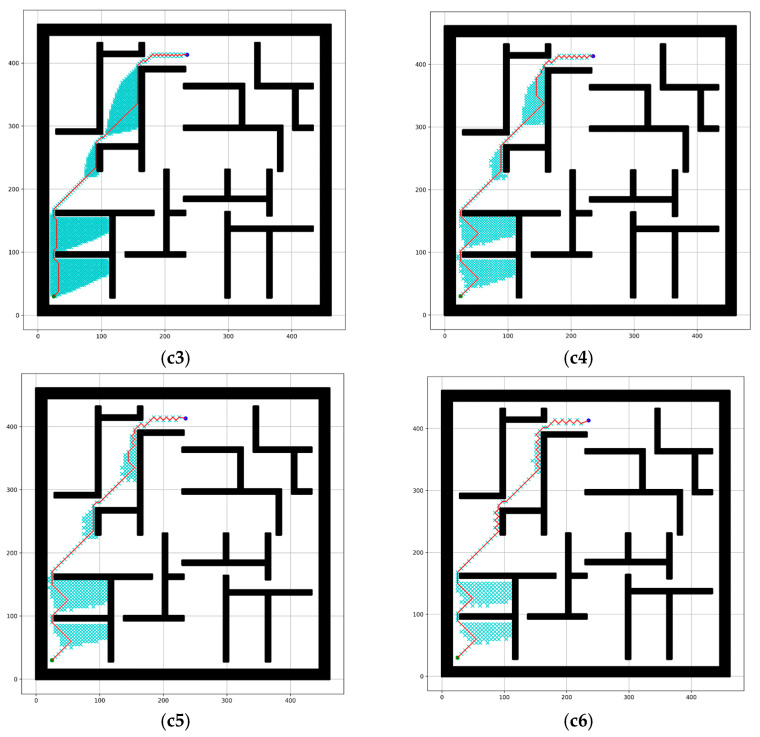
Simulated experimental outcomes of the proposed method with RMBs (n = 1–6) for three types of maps. Here, (**a1**–**a6**), (**b1**–**b6**), and (**c1**–**c6**) present the results for the map types: bugtraq_forest, gaps_and_forest, and mazes. White = free cell, black = obstacle cell, green and blue circle = start and goal point, red line = found path, and cyan cross = cell searched.

**Figure 9:**
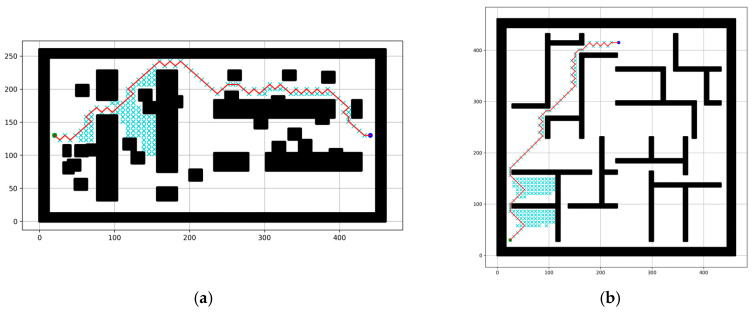
Simulated experimental outcomes of the proposed method with RMB n = 7. Here, (**a**,**b**) present the results for the map types gaps_and_forest and mazes.

The simulated outcomes using the proposed RMBs with different sizes (n = 1–6) for the A* path-planning algorithm are depicted in Figure 8. The RMB size is assumed (n = 1–6) for the experiment because if n increases more, it provides mostly similar results as n = 6, as shown in Figure 8(b6,c6), and Figure 9a,b, which may not affect the results. The resulting parameters in Figure 8(b6) are CS = 814, TC = 0.0197, PC = 570; Figure 9a: CS = 794, TC = 0.0196, PC = 570; Figure 8(c6): CS = 1230, TC = 0.0313, PC = 456; Figure 9b: CS = 1205, TC = 0.0313, PC = 456. They show that the result doesn’t have significant changes.

The experimental results for three types of map dimensions and three of the five map types are shown in Figure 8. (a1–a6), (b1–b6), and (c1–c6) show the result of the bugtraq_forest, gaps_and_forest, and mazes grid maps, where (a1–a6) has a map dimension of 261 × 261, (b1–b6) has 462 × 261, and (c1–c6) has 462 × 462. To find the target point, the number of search nodes drastically decreases with an increase in the size of the proposed RMB. The results for the RMB size n = 1 are shown in (a1,b1,c1), those for n = 2 are shown in (a2,b2,c2), those for n = 3 are shown in (a3,b3,c3), and so on. However, the path cost increased by an insignificant amount.

In Figure 8 and Figure 9, the red line indicates the path that the proposed approach found toward the target node. Furthermore, the searched grid cells are marked by a cyan cross, for which the number decreases when n increases.

**Figure 10:**
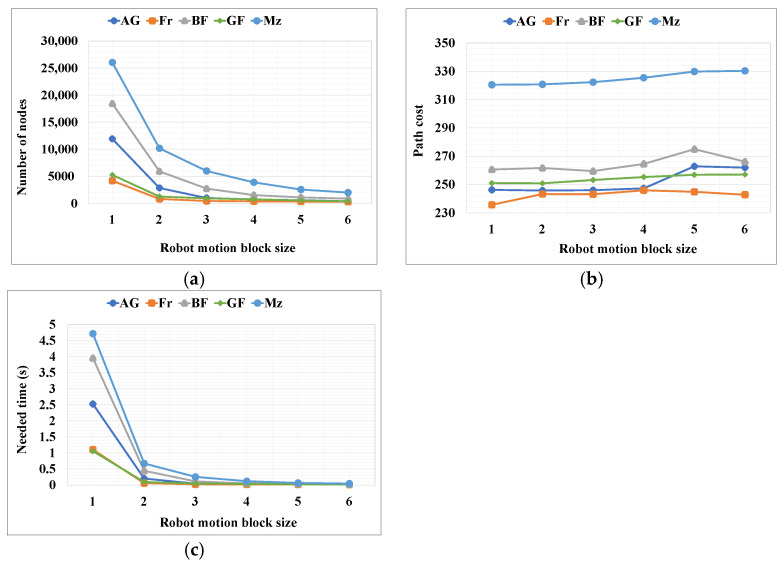
Results for the proposed RMBs for different types of maps where the map dimension was 261 × 261. (**a**) Number of search nodes; (**b**) Path cost; (**c**) Time required for searching the goal node. AG = alternating_gaps, Fr = forest, BF = bugtrap_forest, GF = gaps_and_forest, and Mz = mazes.

**Figure 11:**
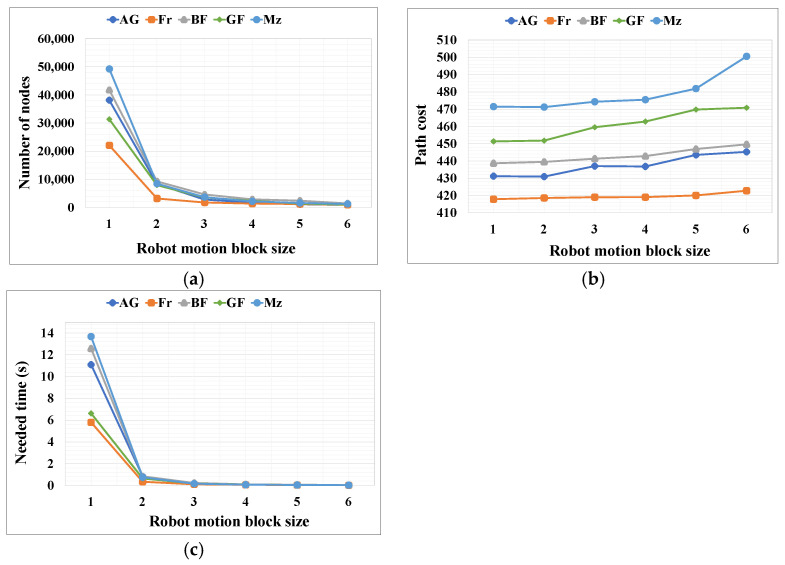
Results for the proposed RMBs for different types of maps where the map dimension was 462 × 261. (**a**) Number of search nodes; (**b**) Path cost; (**c**) Time required for searching the goal node. AG = alternating_gaps, Fr = forest, BF = bugtrap_forest, GF = gaps_and_forest, and Mz = mazes.

**Figure 12:**
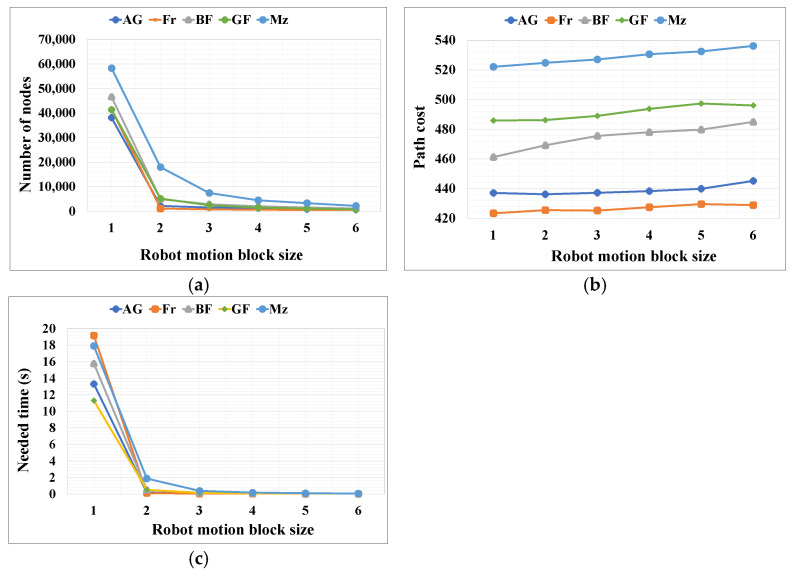
Results for the proposed RMBs for different types of maps the where map dimension was 462 × 462. (**a**) Number of search nodes; (**b**) Path cost; (**c**) Time required for searching the goal node. AG = alternating_gaps, Fr = forest, BF = bugtrap_forest, GF = gaps_and_forest, and Mz = mazes.

For the proposed RMB n = (1–6), three types of data were collected from the experimental results: path cost, number of search nodes, and the time required (seconds) to find the path. Table 3 shows the resultant data for each RMB size and map dimension, which is a mean of all grid maps for each type of map among five types. In addition, a comparison of the outcome results is shown in Figure 10, Figure 11 and Figure 12, where Figure 10a, Figure 11a and Figure 12a show the number of nodes searched, Figure 10b, Figure 11b and Figure 12b show the path cost, and Figure 10c, Figure 11c and Figure 12c show the time required to find the target.

The results in Table 3 and Figure 10, Figure 11 and Figure 12 show that, with an increase in distance for the RMB, the time complexity and number of search nodes to find the target decreases. In contrast, the path cost insignificantly increases because as the search node decreases, the planned path curves around some curved obstacles. As demonstrated by the experimental results in Figure 8, with the increase in RMB size resulting in the decreased number of nodes searched, the robot may not find the straight path towards the goal in some cases, which increases the path cost. Therefore, an optimal RMB selection is essential and is performed using the proposed formula in Equations (5)–(7), as described in Section 3.3.

#### 4.2.3. Results for Optimal Robot Motion Block Selection

**Figure 13:**
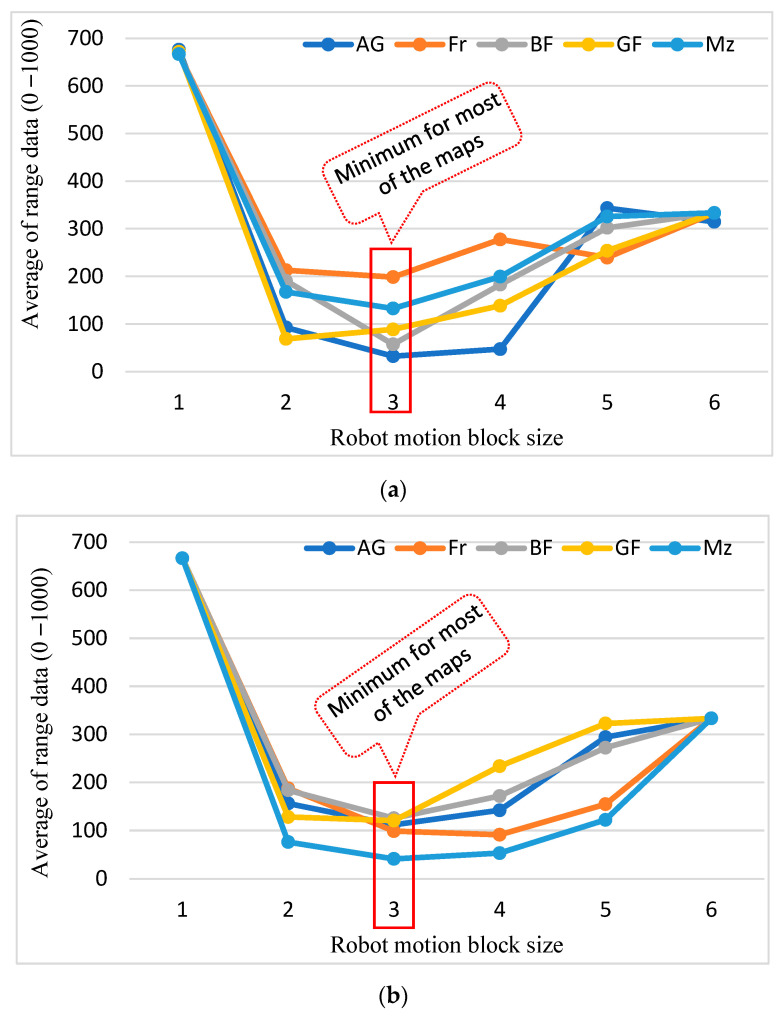

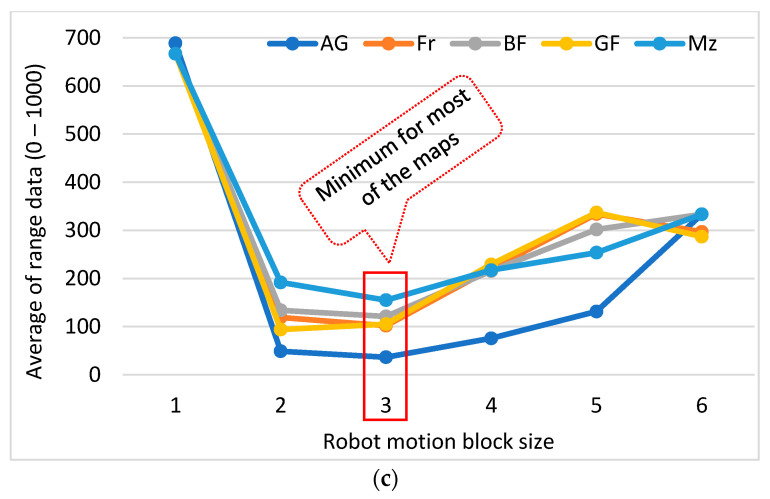
(**a**–**c**) are the experimental results for the proposed optimal RMB based on the experimental data of Table 4. (**a**) For map dimension 261 × 261; (**b**) For map dimension 462 × 261; (**c**) For map dimension 462 × 462.

Table 4 presents the range data for each criterion: the number of search nodes, path cost, and time complexity. Each criterion is weighed equally to achieve the range data and measure the importance. The mean of each map type and map dimension shows that in most cases, the size of RMB n = 3 has the minimum numeric value, and it provides the optimal performance of the A* algorithm. Figure 13 also shows the result for the proposed optimal RMB (which is n = 3). This is because it has the lowest value for both the number of search nodes and time complexity while still having a low value for the path cost. Therefore, it balances the three criteria well and provides an optimal overall performance. It also indicates that for the map type, gaps_and_forest n = 2 is optimal because of its complex and dense obstacle environments. However, the differences between the values of n = 2 and n = 3 are not substantial; for the map dimension 462 × 462 the values are 94.02 and 105.11, respectively.

**Figure 14:**
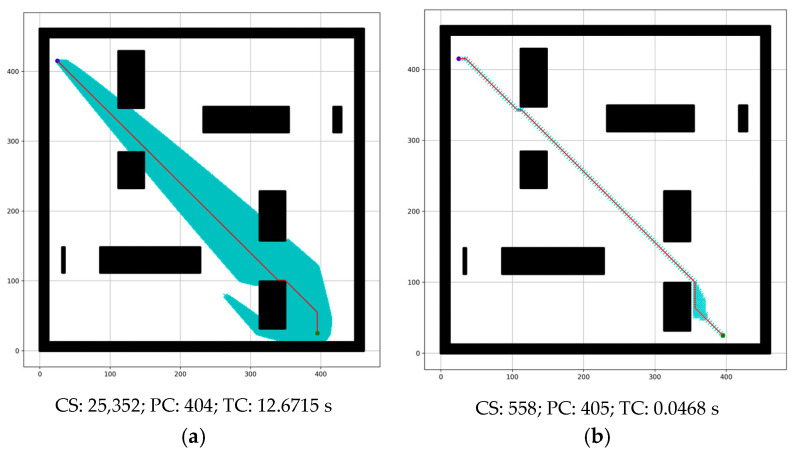
Comparison between conventional (**a**) A* and the (**b**) proposed method in terms of a smaller number of obstacles between the source and destination.

Figure 14 presents comparative results between the proposed method and the conventional A* algorithm and shows that the proposed method excels where there are fewer obstacles between source and destination. The proposed method requires a very small number of nodes to search for the goal, and time complexity reduces significantly compared to the conventional A* algorithm. This is because of the proposed adaptive cost function, which keeps track of the goal node in addition to the proposed optimal RMB. The only challenge is that the path cost increases a little in some cases; for example, in Figure 14, the path cost increases by one.

#### 4.2.4. Comparison of the Proposed Method with the State-of-the-Art Algorithms

**Figure 15:**
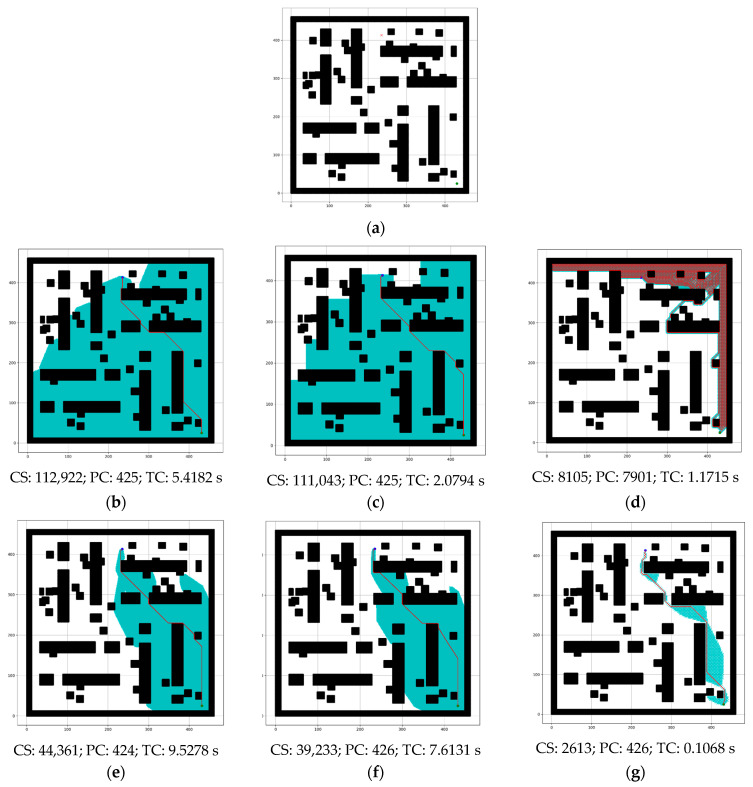
Simulated results for the comparison between different state-of-the-art algorithms and the proposed ORMBA*. (**a**) Grid map showing source and destination; (**b**) Dijkstra; (**c**) BFS; (**d**) DFS; (**e**) Conventional A*; (**f**) TWA*; (**g**) ORMBA*.

Figure 15 shows the resultant figures for different state-of-the-art algorithms on a grid map presented in Figure 15a. In this grid map, the total number of grid cells was 212,521, the number of free cells was 142,483, and the number of obstacle cells was 70,038. The overall performance of Dijkstra and BFS was mostly similar, but the performance of DFS was the worst in terms of path cost. The performance of the turning weight A* (TWA*) [25] was better than the conventional A* algorithm, but the proposed model with optimal RMB (ORMBA*) outperforms all comparison algorithms.

Table 5 represents a comprehensive comparison between the proposed model and the different algorithms, Dijkstra, DFS, BFS, conventional A*, and TWA*. The values from this comparison are the cumulative results collected from the experiment conducted on the considered dataset; thus, these values have fraction numbers. In terms of the number of search nodes and time complexity, the proposed model outperformed the other algorithms. Specifically, first, the number of search nodes for the proposed model is 2824.45, whereas that for the other algorithms ranges from 46,878.88 to 84,677.83. Second, regarding time complexity, the proposed model requires 0.1406 s, whereas the other algorithms require 1.6375 to 16.5898 s. Finally, regarding the path cost, DFS has a value of 4115.53, which is significantly higher than that of the others, and the path cost of the proposed model is mostly similar to that of the remaining algorithms.

**Figure 16:**
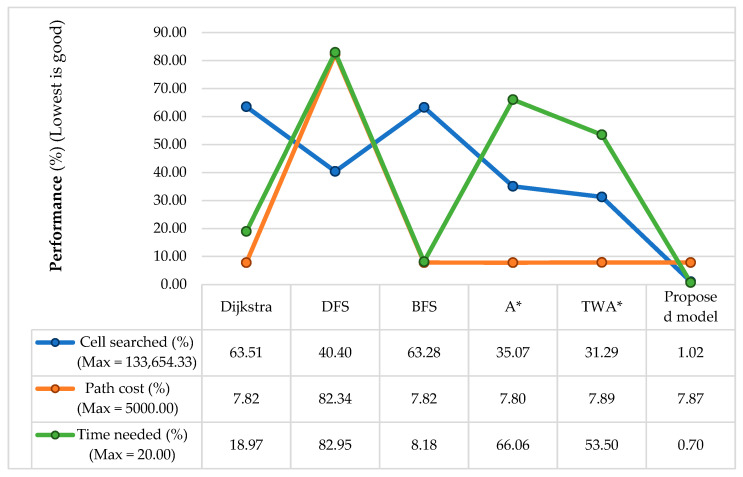
Performance of different algorithms compared with the proposed method.

Figure 16 shows the performance comparison graph of the different algorithms and the proposed model considering the data in Table 5 and Equation (9).

(9) $$PC=\frac{\bar{Av}}{Mx}\times 100,$$

Here,

PC= Performance calculation.
$\bar{Av}\;$= mean of ${Av}_{j}$ from j=1 to 3 (map dimensions).
Mx= Maximum value of each parameter.

In the performance calculation, the considered range for the number of search nodes is 0–133,654.33, as the maximum number of nodes in the dataset is 68,121 for the map dimension 261 × 261, 120,321 for 462 × 261, and 212,521 for 462 × 462. The average of these three values is 133,654.33. Similarly, the considered ranges for the path cost and time complexity are assumed 0–4500 and 0–20 s, respectively. In this graph, the lowest performance percentage indicates good performance as the lowest values represent the lowest number of search nodes, a lower path cost, and a lower time complexity to find the target. The table in Figure 16 shows that the proposed model outperforms the other algorithms in terms of the number of search nodes and time complexity. Its path cost is mostly similar to that of the other algorithms except for DFS, which has the highest path cost.

#### 4.2.5. ROS Experiments

**Figure 17:**
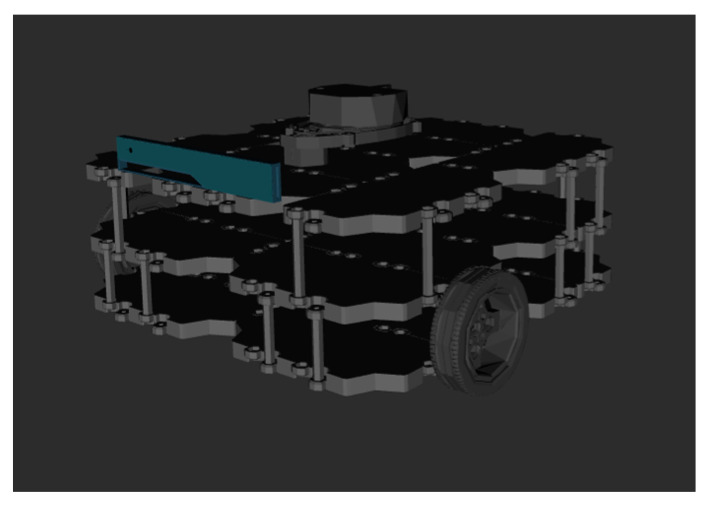
TurtleBot3 Waffle robot in the simulator.

This section presents an experiment for further validation of the proposed method: the ORMBA* and the comparison methods are implemented using the Gazebo Simulator, which uses ROS Gazebo packages on Ubuntu 18.04. To perform this experiment, a TurtleBot3 Waffle three-wheeled robot is employed as an experimental robot and is presented in Figure 17.

**Figure 18:**
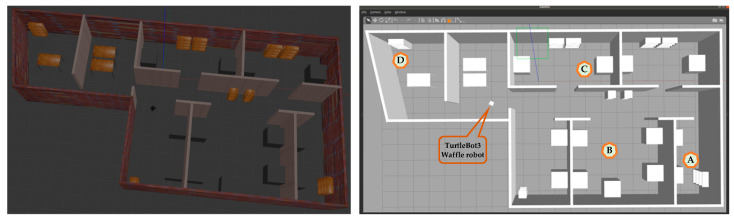
Prepared laboratory environment in the simulator.

In this experiment, firstly, a laboratory environment is prepared in the Gazebo simulator, as shown in Figure 18. The dimensions of the environment are 20 m × 10 m. It contains maze-like walls and different obstacles such as blocks, tables, bookshelves, and others. Four landmarks are selected and considered as source and/or destination for this experiment, as shown in Figure 18. Here, (A) and (B) are the first source and destination, (B) and (C) are the next source and destination, and so on.

**Figure 19:**
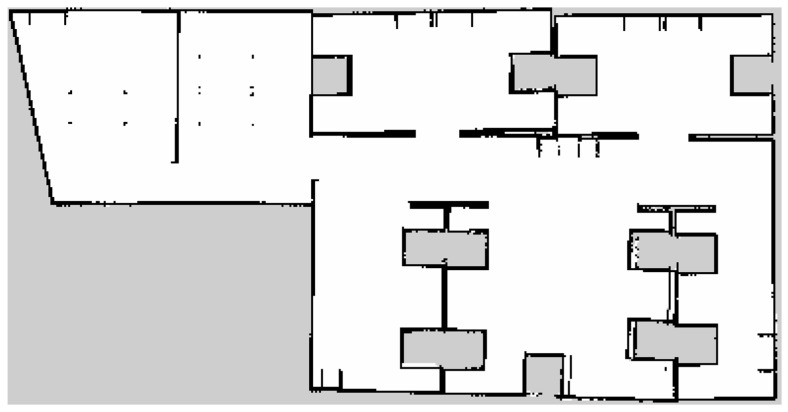
2D laboratory environment constructed using SLAM.

Secondly, the SLAM technique is used to create the 2D construction of the test laboratory environment. The Gmapping method of the SLAM and amcl from the ROS packages is used, which helps the mobile robot to generate a 2D occupancy grid map (similar to a floorplan of a building) and localization by processing lidar information gathered during robot movement. Figure 19 shows the 2D-constructed laboratory environment.

**Figure 20:**
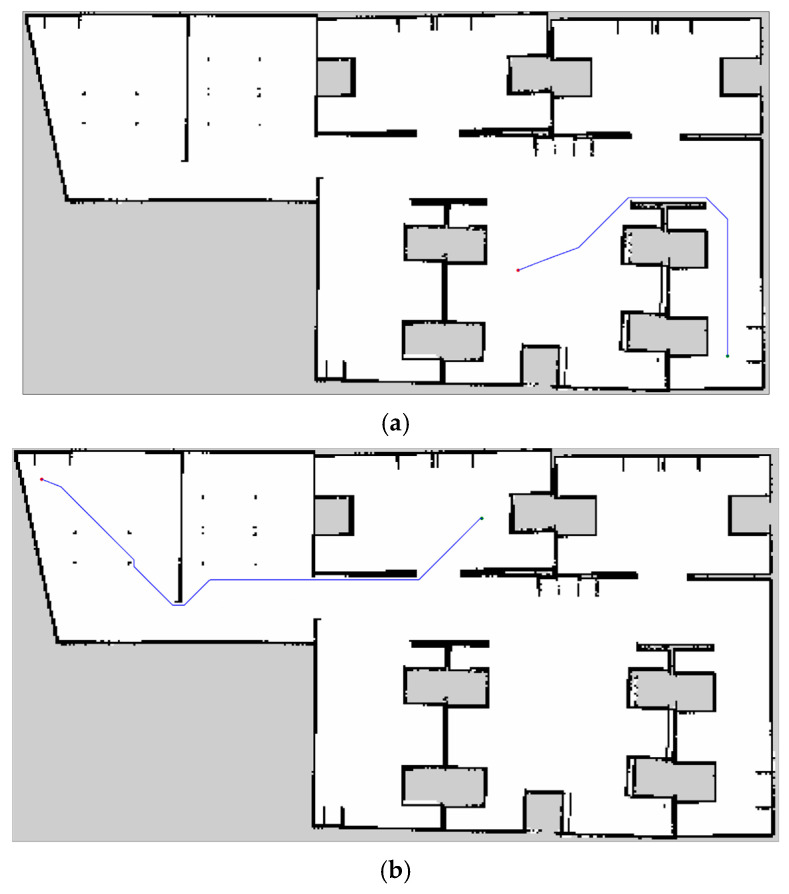
ORMBA* path planning results using the Gazebo simulator and ROS platform. (**a**) Presents the result for source **A** to destination **B** and (**b**) presents the result for **C** to **D** as mentioned in Figure 18. Green and blue circle = start and goal point, blue line = planned path.

Finally, the path-planning methods are used to plan the path and navigate the robot from sources to destinations in the prepared laboratory environment in the simulator. ROS offers different interfaces, including global and local path planners. For this experiment, only global path planning is required based on the destination; hence, the ORMBA* path-planning method was integrated as a new global path planner. This allows ROS to utilize this new planner to operate the robot effectively. For the visualization tool, Rviz is used beside the Gazebo simulator to view the navigation tasks, and the map resolution is set to 0.05. Figure 20 shows the result of the proposed method (ORMBA*).

The usual method to integrate a new path-planning algorithm such as ORMBA* in the ROS platform is to write the code in C++, but for this experiment, Python was used. However, the base packages *global_path_planning*, *srv_clint_plugin*, and *pp_msgs* are required to make the new path-planning algorithm workable. The package *global_path_planning* contains the necessary functionality for a path-planning server and custom algorithm such as ORMBA*. The server receives and processes path planning requests from the client and final planned path returns. Different custom messages defined in *pp_msgs* facilitate the communication. The client program is started by *srv_clint_plugin*, which interfaces with the navigation packages. The client program receives the different parameters, such as the current state, goal node, cost map, and others, and then sends them to the server, which calculates the path to follow. Finally, the client program sends the received planned path to the navigation package to navigate the robot. The Python client library *rospy* for ROS is used. It provides a convenient way to interface with ROS using Python, offering libraries to communicate with ROS topics, services, parameters, and base packages. To control the TurtleBot3, another package, *geometry_msgs*, is used, which defines the angular and linear velocities. Among the ROS topic list, *cmd_vel* is used to publish the control commands.

**Figure 21:**
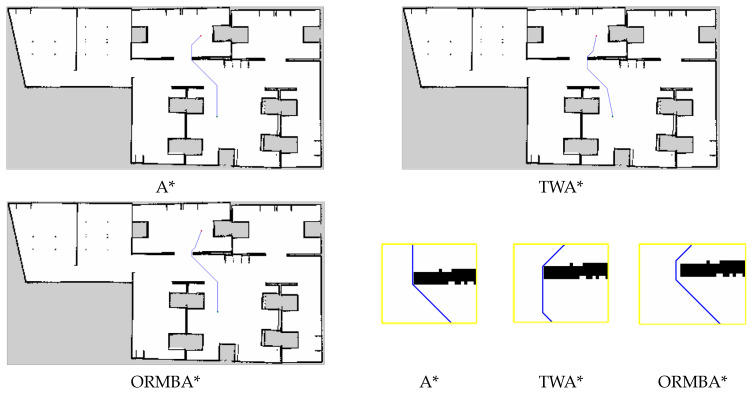
Results of comparison between A*, TWA*, and ORMBA* using the Gazebo simulator and ROS platform for source **B** to destination **C** as mentioned in Figure 18. Green and blue circle = start and goal point, blue line = planned path.

Table 6 and Figure 21 present the performance results of the comparison between A*, TWA*, and ORMBA* methods, which are collected from the Gazebo simulation and ROS platform and the average of all three source-to-destination points. In the performance metrics, the time is calculated using the *rospy.Time* function by calculating the start and end time of the global path planner in the ROS path-planning server. The ROS client program receives the planned path and the length of the planned path from the server. To calculate the length in meters, the path length needs to be multiplied by the map resolution, which is mentioned above.

In this ROS experiment, the robot requires very small search time for the proposed ORMBA* compared to the conventional A* and TWA*, especially when the source-to-destination distance is very large. Also, the robot moves very close to the obstacles in corners for A* and TWA*, which may make it challenging for the robot to move at corners, where the proposed method advances as it keeps a distance from the obstacles, as shown in Figure 21. These advancements help the robot to perform the whole planning and navigation operation much faster and more safely, which is most important for a real-world application.

### 4.3. Discussion

This study aims to enhance the efficiency of the A* path-planning algorithm by minimizing the time complexity and the number of search nodes while maintaining a minimum path cost. To validate the robustness and generalize the proposed ORMBA*, this paper presents the experiment on seven thousand grid maps categorized into five types. Each type has different environmental scenarios with obstacles and three different map sizes. Also, four sets of different sources and destinations were selected for each of the maps. In all the scenarios, the proposed ORMBA* significantly improves in terms of the number of search nodes and time complexity compared to other state-of-the-art algorithms. In light obstacle scenarios, the ORMBA* excels, as presented in Figure 14. In addition, the ROS experiment in a simulated environment presents the improvement and robustness of the proposed ORMBA*.

The experimental results presented in Figure 7 and Table 2 show improved performance compared to the conventional A* algorithm because of its adaptive cost function for the robot movement, which keeps track of the goal position. The reduction in time complexity, the number of search nodes, and path cost are approximately 29.05%, 32.58%, and 0.04%. According to Table 3 and Figure 10, Figure 11 and Figure 12, increasing the size of the proposed RMB reduces the time complexity and the number of search nodes significantly. Figure 8 samples the simulation results. The path costs increased slightly for the increased size of RMB; however, the time complexity and the number of search nodes decreased. Thus, the proposed model selects the optimal RMB to apply in different applications. The experimental results for the optimal RMB selection are presented in Table 4 and Figure 13. According to the experimental results, the RMB n = 3 is optimal as it provides balanced, improved results in most cases, as shown in Figure 13. An additional experiment was conducted where the proposed model was compared with other similar algorithms, as shown in Table 5 and Figure 15 and Figure 16. The experimental results show that the proposed model outperforms the comparison algorithms. The TWA* performed better than the conventional A* algorithm and other algorithms, whereas the proposed model ORMBA* outperformed by decreasing the number of search nodes by 93.98% and 93.24% and the time complexity by 98.94% and 98.64% compared to the conventional A* and TWA* algorithms, calculated using Equation (8). The path cost of the proposed model increased by 0.82% from A* and decreased by 0.32% from TWA*, which is comparable to other algorithms except for DFS, which had the highest path cost. Overall, the proposed method shows significant improvement in terms of the number of search nodes and time complexity by keeping the path cost almost similar to that of state-of-the-art algorithms. In some cases, the proposed model shows an insignificantly increased path cost, which may not have a large impact, but the other criterion of improvement has a significant impact on robot navigation. The proposed method also performed well in the ROS experiments, as presented in Figure 21 and Table 6.

## 5. Conclusions

Path planning is crucial for autonomous robot navigation, allowing robots to find optimal routes while avoiding obstacles. A* is a popular global path-planning algorithm due to its heuristic approach. However, conventional A* has limitations, such as computational complexity and extensive node searching in the grid spaces. Improvement of such issues may increase the usability of this path-planning algorithm for autonomous robot applications in dynamic real-world environments. To consider this improvement, this paper proposes an optimal RMB with an adaptive cost function for the A* algorithm that significantly improves its overall performance. In this approach, firstly, the robot movement costs are calculated by the proposed adaptive cost function, which keeps track of the goal to reduce the number of searches to find the goal. Secondly, using the optimal RMB, the robot checks a certain distance in eight surrounding directions each time and puts only the end node in the OPEN list of the A* algorithm. To select the next movement from the OPEN list, it applies a heuristic approach. This approach significantly reduces the number of search nodes and time complexity while achieving an acceptable path cost. To evaluate the proposed approach, an open-source dataset with seven thousand grid maps, categorized into five types, is used. To enhance the complexity of the dataset, three different map sizes and sets of source-to-destination points are introduced. This large, customized dataset helps to evaluate the efficiency, robustness, and scalability of the proposed model. A comprehensive experiment is conducted to validate the performance and compare it to the state-of-the-art algorithms. The experimental results show the effectiveness of the proposed ORMBA* by improving performance by approximately 93% for the number of search nodes and 95% for the time complexity. Additionally, the experiment on the ROS platform shows the robot navigation improvement in a simulated laboratory environment.

## Figures and Tables

**Table 1:** Summary of related works in the area of path-planning methods that investigate concrete ways to show the performance improvement.

Article	Test Environment with Obstacles	Cost Function Optimization	RMB Improvement	Test on Robots with Sensors	ROS Experiment	Test on Different Map Sizes	Test on Large Numbers of Maps
[13]	√	-	-	√	√	-	-
[16]	√	-	-	-	-	-	-
[21]	√	√	-	-	-	√	-
[25]	√	√	-	√	√	-	-
[27]	√	-	√	√	√	-	-
Proposed method	√	√	√	√	√	√	√

**Table 2:** Experimental results for the comparison between conventional A* and the proposed method with RMB *n* = 1.

Algorithms	# Search Nodes	Path Cost	Time Required (s)
Conventional A*	46,878.88	390.10	13.2112
Proposed model	**31,604.34**	**389.94**	**9.3736**

**Table 3:** Experimental results for the proposed method with different RMB sizes.

Map Type	Map Dimension	Parameters	RMB					
			*n* = 1	*n* = 2	*n* = 3	*n* = 4	*n* = 5	*n* = 6
alternating_gaps	261 × 261	# search nodes	11,903.92	2838.90	1198.65	903.76	786.34	502.14
		Path cost	246.26	245.78	246.01	247.32	262.92	261.95
		Time required (s)	2.5259	0.2006	0.0735	0.0585	0.0296	0.0166
	462 × 261	# search nodes	38,132.17	8351.43	2789.37	1702.49	1076.50	888.37
		Path cost	431.22	433.96	435.06	436.84	443.55	445.29
		Time required (s)	11.0979	0.8327	0.1473	0.0712	0.0383	0.0221
	462 × 462	# search nodes	38,069.77	3958.41	1522.72	1266.83	811.02	632.05
		Path cost	437.09	436.52	437.20	438.33	439.89	445.18
		Time required (s)	13.3132	0.7858	0.1109	0.0383	0.0245	0.0202
forest	261 × 261	# search nodes	8181.34	1809.13	1142.62	869.21	629.31	482.98
		Path cost	235.69	241.27	242.17	245.92	244.84	248.83
		Time required (s)	1.9034	0.0982	0.0521	0.0256	0.0245	0.0195
	462 × 261	# search nodes	22,056.06	3855.53	1764.34	1356.10	1175.00	936.63
		Path cost	417.87	419.61	419.07	419.09	420.10	422.81
		Time required (s)	5.8014	0.4410	0.1067	0.0617	0.0336	0.0200
	462 × 462	# search nodes	41,497.96	1098.45	759.14	632.76	567.44	495.21
		Path cost	423.38	425.47	425.23	427.45	429.58	428.90
		Time required (s)	19.1736	0.1115	0.0405	0.0245	0.0186	0.0156
bugtrap_forest	261 × 261	# search nodes	18,436.83	5909.82	2731.87	1533.71	1105.00	892.15
		Path cost	260.13	261.72	260.52	264.59	268.00	268.97
		Time required (s)	3.9576	0.4435	0.1137	0.0500	0.0383	0.0245
	462 × 261	# search nodes	41,725.85	9255.84	4571.77	2855.64	2372.93	1382.53
		Path cost	436.70	440.48	440.36	442.83	446.93	449.65
		Time required (s)	12.5779	0.8432	0.2342	0.1087	0.0692	0.0383
	462 × 462	# search nodes	46,582.38	5011.74	1913.54	1509.35	1339.76	1121.14
		Path cost	460.24	465.91	467.55	474.02	479.76	481.96
		Time required (s)	15.7666	0.8953	0.1778	0.0861	0.0588	0.0291
gaps_and_forest	261 × 261	# search nodes	9260.30	1858.17	938.19	761.81	558.93	380.48
		Path cost	251.04	250.87	253.21	255.33	259.90	263.07
		Time required (s)	2.3573	0.1129	0.0455	0.0334	0.0206	0.0186
	462 × 261	# search nodes	31,361.01	7935.98	3511.17	2356.03	1336.57	991.89
		Path cost	448.15	449.54	453.95	462.83	469.79	470.84
		Time required (s)	6.6211	0.6473	0.1781	0.0847	0.0411	0.0245
	462 × 462	# search nodes	41,334.98	5584.34	2427.80	1517.65	1124.17	759.52
		Path cost	485.89	486.88	488.99	493.71	497.64	496.01
		Time required (s)	11.3063	0.9169	0.1356	0.0595	0.0393	0.0208
mazes	261 × 261	# search nodes	26,045.38	10,176.25	5986.73	3896.61	2564.64	2008.82
		Path cost	320.51	320.78	322.35	325.45	329.83	330.33
		Time required (s)	4.7198	0.6722	0.2536	0.1198	0.0658	0.0435
	462 × 261	# search nodes	49,235.53	8426.30	3654.47	2122.42	1590.48	1247.94
		Path cost	471.47	472.21	473.32	475.49	481.91	500.58
		Time required (s)	13.6747	0.7576	0.1655	0.0679	0.0479	0.0346
	462 × 462	# search nodes	58,241.65	17,939.49	7454.32	4472.24	3274.23	2194.61
		Path cost	522.04	524.76	527.02	530.56	532.45	536.13
		Time required (s)	17.9080	1.8732	0.3839	0.1729	0.1012	0.0563

**Table 4:** Experimental results to select the optimal RMB using Equations (5)–(7).

Map Type	Map Dimension	Parameters	RMB					
			n = 1	n = 2	n = 3	n = 4	n = 5	n = 6
alternating_gaps	261 × 261	# search nodes	1000.00	204.95	61.09	35.22	24.93	0.00
		Path cost	28.30	0.00	13.42	89.74	1000.00	943.69
		Time required	1000.00	73.30	22.66	16.67	5.16	0.00
		*Average*	676.10	92.75	**32.39**	47.21	343.36	314.56
	462 × 261	# search nodes	1000.00	200.38	51.04	21.86	5.05	0.00
		Path cost	0.00	194.97	273.07	399.64	876.12	1000.00
		Time required	1000.00	73.18	11.31	4.44	1.46	0.00
		*Average*	666.67	156.18	**111.80**	141.98	294.21	333.33
	462 × 462	# search nodes	1000.00	88.85	23.79	16.96	4.78	0.00
		Path cost	66.33	0.00	79.17	208.90	389.30	1000.00
		Time required	1000.00	57.59	6.82	1.36	0.32	0.00
		*Average*	688.78	48.81	**36.59**	75.74	131.47	333.33
forest	261 × 261	# search nodes	1000.00	172.27	85.69	50.17	19.01	0.00
		Path cost	0.00	424.75	493.11	779.07	696.73	1000.00
		Time required	1000.00	41.75	17.29	3.24	2.65	0.00
		*Average*	666.67	212.92	**198.70**	277.49	239.46	333.33
	462 × 261	# search nodes	1000.00	138.21	39.19	19.86	11.29	0.00
		Path cost	0.00	351.94	241.79	246.73	450.17	1000.00
		Time required	1000.00	72.83	15.00	7.21	2.35	0.00
		*Average*	666.67	187.66	**98.66**	91.27	154.61	333.33
	462 × 462	# search nodes	1000.00	14.71	6.44	3.35	1.76	0.00
		Path cost	0.00	337.47	298.24	656.74	1000.00	890.39
		Time required	1000.00	5.00	1.30	0.46	0.15	0.00
		*Average*	666.67	119.06	**101.99**	220.18	333.97	296.80
bugtrap_forest	261 × 261	# search nodes	1000.00	285.99	104.86	36.57	12.13	0.00
		Path cost	0.00	180.21	44.58	504.79	890.38	1000.00
		Time required	1000.00	106.54	22.70	6.50	3.51	0.00
		*Average*	666.67	190.91	**57.38**	182.62	302.01	333.33
	462 × 261	# search nodes	1000.00	195.16	79.05	36.51	24.55	0.00
		Path cost	0.00	291.88	282.17	472.96	789.82	1000.00
		Time required	1000.00	64.19	15.63	5.61	2.46	0.00
		*Average*	666.67	183.74	**125.62**	171.70	272.28	333.33
	462 × 462	# search nodes	1000.00	85.58	17.43	8.54	4.81	0.00
		Path cost	0.00	260.96	336.57	634.61	898.72	1000.00
		Time required	1000.00	55.04	9.45	3.63	1.89	0.00
		*Average*	666.67	133.86	**121.15**	215.59	301.80	333.33
gaps_and_forest	261 × 261	# search nodes	1000.00	166.41	62.81	42.94	20.10	0.00
		Path cost	14.27	0.00	191.82	365.81	740.76	1000.00
		Time required	1000.00	40.32	11.52	6.36	0.85	0.00
		*Average*	671.42	**68.91**	88.72	138.37	253.90	333.33
	462 × 261	# search nodes	1000.00	228.66	82.96	44.92	11.35	0.00
		Path cost	0.00	60.99	255.46	647.02	953.69	1000.00
		Time required	1000.00	94.41	23.30	9.13	2.53	0.00
		*Average*	666.67	128.02	**120.57**	233.69	322.52	333.33
	462 × 462	# search nodes	1000.00	118.91	41.12	18.68	8.99	0.00
		Path cost	0.00	83.76	264.05	665.66	1000.00	860.81
		Time required	1000.00	79.40	10.17	3.43	1.64	0.00
		*Average*	666.67	**94.02**	105.11	229.26	336.87	286.94
mazes	261 × 261	# search nodes	1000.00	339.79	165.49	78.54	23.12	0.00
		Path cost	0.00	27.82	187.67	503.39	949.12	1000.00
		Time required	1000.00	134.45	44.93	16.32	4.78	0.00
		*Average*	666.67	167.35	**132.70**	199.42	325.67	333.33
	462 × 261	# search nodes	1000.00	149.59	50.15	18.22	7.14	0.00
		Path cost	0.00	25.29	63.31	138.00	358.44	1000.00
		Time required	1000.00	53.01	9.60	2.44	0.97	0.00
		*Average*	666.67	75.96	**41.02**	52.89	122.18	333.33
	462 × 462	# search nodes	1000.00	280.92	93.84	40.64	19.26	0.00
		Path cost	0.00	192.96	353.21	604.99	739.05	1000.00
		Time required	1000.00	101.78	18.35	6.53	2.52	0.00
		*Average*	666.67	191.89	**155.14**	217.39	253.61	333.33

**Table 5:** Experimental results for the comparison between different state-of-the-art algorithms and the proposed ORMBA*.

Algorithms	Map Dimension	Number of Search Nodes	Path Cost	Time Required (s)
Dijkstra	261 × 261	43,440.25	258.95	1.5023
	462 × 261	73,496.51	436.65	2.4945
	462 × 462	137,696.72	477.27	7.3836
	*Average*	84,877.83	390.96	3.7935
DFS	261 × 261	36,553.30	1342.70	4.5641
	462 × 261	48,019.90	5644.20	9.5305
	462 × 462	77,399.00	5363.70	35.6750
	*Average*	53,990.73	4116.87	16.5898
BFS	261 × 261	43,874.00	258.40	1.0516
	462 × 261	72,871.50	436.65	1.2953
	462 × 462	136,987.80	477.70	2.5602
	*Average*	84,577.77	390.92	1.6357
A*	261 × 261	22,587.45	257.95	4.5156
	462 × 261	49,740.21	435.65	12.8063
	462 × 462	68,308.97	476.70	22.3117
	*Average*	46,878.88	**390.10**	13.2112
TWA*	261 × 261	19,218.59	262.32	3.9392
	462 × 261	44,035.81	442.96	9.8472
	462 × 462	62,199.15	478.47	18.3117
	*Average*	41,817.85	394.58	10.6993
**Proposed model** (ORMBA*)	261 × 261	2219.61	264.85	0.0957
	462 × 261	3258.22	444.35	0.1664
	462 × 462	2995.51	470.80	0.1597
	*Average*	**2824.45**	393.33	**0.1406**

**Table 6:** Performance metrics in the Gazebo simulation and ROS platform.

Parameters		Source to Destination	A to B	B to C	C to D	Average
	Algorithms					
Path length (m)	A*		42.95	24.15	54.80	**40.63**
	TWA*		43.10	24.25	54.93	40.75
	ORMBA*		43.08	24.23	54.90	40.74
Search time (s)	A*		18.7923	2.9738	114.5114	45.4258
	TWA*		17.0025	2.7609	102.3118	40.6917
	ORMBA*		3.9341	0.5793	20.5904	**8.3679**

## Data Availability

Data are contained within the article.
